# Predictive prioritization of genes significantly associated with biotic and abiotic stresses in maize using machine learning algorithms

**DOI:** 10.3389/fpls.2025.1611784

**Published:** 2025-06-19

**Authors:** Anjan Kumar Pradhan, Prasad Gandham, Kanniah Rajasekaran, Niranjan Baisakh

**Affiliations:** ^1^ School of Plant, Environmental and Soil Sciences, Louisiana State University Agricultural Center, Baton Rouge, LA, United States; ^2^ Southern Regional Research Center, USDA-ARS, New Orleans, LA, United States

**Keywords:** a(biotic) stress, artificial intelligence, gene expression, maize, RNA-Seq

## Abstract

Both biotic and abiotic stresses pose serious threats to the growth and productivity of crop plants, including maize worldwide. Identifying genes and associated networks underlying stress resistance responses in maize is paramount. A meta-transcriptome approach was undertaken to interrogate 39,756 genes differentially expressed in response to biotic and abiotic stresses in maize were interrogated for prioritization through seven machine learning (ML) models, such as support vector machine (SVM), partial least squares discriminant analysis (PLSDA), k-nearest neighbors (KNN), gradient boosting machine (GBM), random forest (RF), naïve bayes (NB), and decision tree (DT) to predict top-most significant genes for stress conditions. Improved performances of the algorithms via feature selection from the raw gene features identified 235 unique genes as top candidate genes across all models for all stresses. Three genes such as *Zm00001eb176680*, *Zm00001eb176940*, and *Zm00001eb179190* expressed as *bZIP* transcription factor 68, glycine-rich cell wall structural protein 2, and aldehyde dehydrogenase 11 (*ALDH11*), respectively were commonly predicted as top-most candidates between abiotic stress and combined stresses and were identified from a weighted gene co-expression network as the hub genes in the brown module. However, only one gene *Zm00001eb038720* encoding RNA-binding protein AU-1/Ribonuclease E/G, predicted by the PLSDA algorithm, was found commonly expressed under both biotic and abiotic stress. Genes involved in hormone signaling and nucleotide binding were significantly differentially regulated under stress conditions. These genes had an abundance of antioxidant responsive elements and abscisic acid responsive elements in their promoter region, suggesting their role in stress response. The top-ranked genes predicted to be key players in multiple stress resistance in maize need to be functional validated to ascertain their roles and further utilization in developing stress-resistant maize varieties.

## Introduction

Plants are constantly subjected to various biotic and abiotic stresses that have negative effects on the growth, development, and productivity of economically important crops including maize (*Zea mays* L.) (Ramegowda and Senthil-Kumar, 2015). Maize is a main grain, forage, and energy crop as well as a genetic model plant (Farooqi et al., 2022). It is one of the most important cereal crops cultivated worldwide, mainly in Africa and South America (Kimotho et al., 2019). After wheat and rice, maize is the most frequent cereal food in Mexico (Nazari et al., 2023). United States remains the largest producer of maize. According to USDA, corn production for the 2024/25 marketing year is projected to be around 377.63 million metric tons, a 1% reduction than last year, which is attributed to extreme drought and heat during the 2024 crop year (https://www.fas.usda.gov/data/production/commodity/0440000).

Abiotic stresses such as drought, cold, submergence, salinity, waterlogging, heavy metal contamination, or nutrient deficiency can reduce crop yield by more than >50% (Mallikarjuna et al., 2020). Similarly, biotic stresses, caused by living organisms such as bacteria, viruses, fungi, or nematodes, negatively affect the productivity of maize by approximately 10% (Nazari et al., 2023). Plants undergo genome-wide transcriptome reprogramming in response to external stressors, which leads to induction and/or repression of genes associated with various mechanisms at whole plant, organellar, cellular, and molecular levels. Recent advances in various omics technologies have accelerated the identification of genes and biological processes controlling stress responses in plants. Analysis of transcriptomes has made it possible to identify genes overexpressed/repressed under specific stress (Sharma et al., 2013). However, in field conditions, plants are repeatedly exposed to multiple stresses simultaneously, which requires the plants to exercise efficient molecular mechanisms to recognize a host of signals to effectively respond to more than one stress (Sharma et al., 2013). Both biotic and abiotic stress factors and their various combinations in natural conditions elicit modified stress responses in plants. In addition to several genes, transcription factors (TFs) are known to be significantly involved in stress response in plants (Zhu, 2002). Many TFs from AP2/IREP, bZIP, MYB, NAC, and WRKY families have been found to improve stress resistance by regulating the expression of other stress-responsive genes in plants (Qin et al., 2011). Thus, the identification and characterization of key genes that are co-expressed in plants’ response to both abiotic and biotic stresses would provide targets for genetic strategies to improve stress tolerance.

Over the years, several stress-regulated genes have been identified in maize using both microarray and RNA-seq approaches (Hayford et al., 2024). However, deciphering unique genes responding to specific or multiple stresses requires significant computational maneuvers. Meta-analysis is recognized as a reasonable yet statistically powerful approach where the results from multiple independent studies can be combined to eliminate the challenges due to variations between individual studies (Ramasamy et al., 2008; Keel and Lindholm-Perry, 2022). Meta-analysis has been used successfully on transcriptomic data of several crops including maize to identify potentially top candidate genes that are regulated in plants to cope with stress (Baisakh et al., 2023; Hayford et al., 2024; Wang et al., 2022; Nazari et al., 2024).

The multidimensionality of RNA-seq and microarray data owing to the high number of variables and genes with minimal sample size necessitates a gene selection technique to find the most informative, expressed genes and remove the redundancy in the original space (Mahendran et al., 2020). Machine Learning (ML), which is a subset of artificial intelligence, focuses on training the algorithms based on available datasets thus enabling models to learn to make decisions on their own from data without explicit programming. ML uses feature extraction and selection as a reduction technique for classification performance to make decisions on the top features (Kira and Rendell, 1992). In this study, we conducted a meta-analysis for integrating RNA-seq data across independent studies on biotic and abiotic stresses in maize to predictably identify the top-most significantly differentially expressed genes using ML tools. Furthermore, we compared the results from multiple ML models and integrated gene co-expression network analysis to identify the most useful stress-responsive genes.

## Materials and methods

### RNA-seq data collection and feature counts

All RNA-seq datasets related to both biotic and abiotic stresses in maize (**Supplementary Table 1**) were searched online using the publicly available sequence repository database i.e., NCBI Gene Sequence Read Archive (https://www.ncbi.nlm.nih.gov/sra). Raw sequence reads were subjected to quality control and filtering following the method described earlier (Bedre et al., 2015). Clean sequence reads were mapped against the B73 reference genome NAM 5.0 using HISAT2 (Kim et al., 2019) with default parameters. Mapped reads were then counted for genomic features such as genes, chromosomal locations, etc. using the FeatureCounts program (Liao et al., 2014).

### Read counts preprocessing and merging

Gene expression data are often associated with high inconsistency due to noise and pieces attributed to the differences in sample numbers, labels, experimental conditions, etc. To correct these biases caused by non-biological conditions, data were normalized using “normalize.quantiles” in R package *preprocessCore* version 1.56.0 following Schadt et al. (2001). However, normalization procedures do not adjust the sample data for batch effects (BE) when merging batches of data from multiple experiments that contain large batch-to-batch variation. Therefore, BE correction was performed using the ‘*ComBat*’ function within the SVA package in R as described earlier (Zhang et al., 2022), which uses empirical Bayes method that estimates the LS parameters (mean and variance) for each gene and merges information from multiple genes with similar expression attributes in each batch.

### Machine learning gene selection approaches

Gene expression data in the form of classified attributes with feature counts of several thousands of genes with expression patterns from multiple stress samples were analyzed to identify the most important genes, which required extraction and selection of features with discriminating ability. For ML, 80% of the data was used as a training set and the remaining 20% as the test/validation set. The classification of maize samples for the gene expression under control and (a)biotic stress was used to select the ML models that can best identify the top stress-responsive genes. We used seven ML algorithms (i.e., SVM, support vector machine; PLSDA, partial least squares discriminant analysis, KNN: K-nearest neighbors, GBM, gradient boosting machine; RF, random forest; NB, naïve bayes; and DT, decision tree) for the identification of most informative genes from the differentially expressed genes (DEGs). Variable importance (VarImp) evaluation functions were grouped with and without the model information. A model-based approach is more closely tied to its performance, which can integrate the correlation structure between the predictors (genes in this case) into the importance calculation. Each gene is assigned a separate variable importance for each class in classification models where all importance measures are scaled at minimum and maximum values of 0 and 100, respectively. The area under the receiver operating characteristic (ROC) curve (AUC) values for each gene was obtained using the “*filterVarImp”* function.

The SVM algorithm was used to identify top genes with R package *e1071* version 1.7–6 using the function of SVM-radial and default code: RFE (x = data, y = as.numeric (as.factor (group)), sizes = c (seq (2, 40, by = 2)), RFE-Control = rfeControl (functions = caretFuncs, method = “cv”), methods = “svmRadial”). Another model, Random Forest (version 4.6–14), was implemented using the *RF* algorithm with the following parameters: ntree = 100–500 and mtry = 1 – 8 (Kim et al., 2022). The relevance score and ranking of the genes in RF and SVM were determined following the recursive feature elimination (REF) method as per the program manual.

The PLS-DA method was implemented in R package *PLSDA* with PLS regression where Y is a set of binary response variables describing the categories (control or stress) of a categorical variable on X, where X is the gene expression matrix using the equation of Pérez-Enciso and Tenenhaus (2003) that used the algorithm of Wold et al. (1983) to allow for missing values. We identified the top genes using variable importance in projection (*VIP*) for each gene (Eriksson et al., 1999). GBM, an ensemble method (Hastie et al., 2009) is also used for regression and classification methods with reduced variance and bias in simple prediction models (Hastie et al., 2009). The *caret* package in R was used with the GBM function for selecting the top genes. The *caret* package was also used for KNN model with 10-fold cross-validation and default parameter *tune-Length* =10 to select the top-ranked genes for different stresses. Similarly, the DT model used the R package *Rpart* to select the top genes through *mtree* function. Another method, Naïve Bayes version 0.9.7 was adopted for the NB algorithm with 10-fold cross-validation for identifying the most important genes. The performance of each ML model was determined by classification metrics such as accuracy, precision, specificity, sensitivity (recall), F1-score, Mathews correlation coefficient (MCC), and ROC derived from the confusion matrix following the equations described in Sabanci et al. (2022). The confusion matrix was prepared with the maize gene under control labeled as 1 and stress as -1.

### Gene co-expression network analysis

Weighted gene co-expression network analysis (WGCNA) is used in systems biology to make clusters (modules) of highly correlated genes based on the module eigengene (ME) and to identify an intramodular hub gene. The expression values of genes after normalization and batch effect correction were used in the R package *WGCNA* version 1.66 (Langfelder and Horvath, 2008) for weighted co-expression network construction where the similar matrix between each pair of genes across all samples was evaluated based on the Pearson’s correlation values. Modules were identified by the *blockwiseModules* function of the *WGCNA* package with default parameters and a tree cut height of 0.4. The modules were defined at a cut height of 0.98 and a size at 30. Similar modules were merged when dissimilarity of module eigengenes was <0.25. The function *signedKME* was used to calculate module eigengene values (KME) based on the correlation of the module eigengene (ME) with the corresponding gene. Modules with correlation value (r) >0.8 between genes and P-value <0.01 were considered as significant modules (de Silva et al., 2022). Gene significance was calculated based on the *p* value of the linear regression between the gene expression profile with multiple stress conditions. The hub genes in a module were identified based on gene significance value (GS) >0.5, module eigengene (KME) >0.8, and with maximum connections with other genes (Baisakh et al., 2023). Gene co-expression network was visualized using Cytoscape (version 3.10.3) software in R (Shannon et al., 2003; Baisakh et al., 2023).

### Gene ontology and promoter analysis

The ontology of the genes was assigned by the singular enrichment analysis within AgriGO version 2 (https://systemsbiology.cau.edu.cn/agriGOv2/) and gene ontology enrichment was performed using Fisher’s *t-test* (P<0.05) and FDR correction by the Hochberg method. Metabolic pathway enrichment analysis was performed using the DAVID tool version 6.7 (https://davidbioinformatics.nih.gov/tools.jsp). The top genes common between two or more models were used to extract 2000 bp upstream flanking region of sequence using the Ensembl Plants database (http://plants.ensembl.org). Promoter prediction and identification of cis-regulatory elements (CREs) within a promoter were performed using PlantCARE (https://bioinformatics.psb.ugent.be/webtools/plantcare/html/).

## Results and discussion

### DEGs under biotic and abiotic stress

Raw sequence reads of a total of 3,052 samples, which included 976 from biotic (bacteria, fungus, insect, nematode, and weed) and 2,076 from abiotic (drought, heat, cold, waterlogging, salt, nutrient, and mechanical wounding) stresses, were obtained from 52 RNA-seq studies conducted with 12 stress conditions of which five were biotic and seven were abiotic (**Supplementary Table 1**). Finally, 39,756 differentially expressed genes from 1,452 samples (451 from biotic and 1001 from abiotic) and 22 of 52 studies after normalization and batch effect correction, respectively were used in ML models sets to identify the topmost significant genes.

### Identification of top stress-responsive genes by ML models

Identifying top stress-responsive DEGs was conducted for biotic or abiotic stress individually and in combination to identify top genes unique to a specific stress category and common between the stress categories.

#### Prediction of top genes under biotic stress

Based on the confusion matrix (**Supplementary Table 2**) of the seven ML models, SVM performed the best with the highest average accuracy of 76.6% followed by RF (73.0%) and GBM (71.8%) whereas NB, KNN, DT, and PLSDA performed very poorly with an average accuracy of 57% (**Table 1A**). SVM also had the highest specificity (0.81) and MCC (0.54) with precision (0.81) behind KNN (0.84) and sensitivity (0.73) behind NB (0.83), RF (0.80), and GBM (0.75). Interestingly, SVM had the lowest F1-score (0.56). However, MCC, which considers all four parameters in the confusion matrix, is considered a better performance matrix as compared to F1-score, which only considers precision/recall. DT had the worst performance in terms of accuracy (53.5%), sensitivity (0.58), and MCC (0.03), although it had a slightly better specificity over PLSDA and NB, higher precision than NB, and higher F1-score than SVM. DT was the least sensitive algorithm correctly detecting only 0.58 for positive samples.

**Table 1A:** Confusion matrices of machine learning algorithms used with gene expression values under biotic stress in maize.

Models	Accuracy	Specificity	Sensitivity	Precision	F1-score	MCC	FP Rate	ROC Area	PRC Area
NB	57	0.418	0.837	0.45	0.585	0.318	0.581	1.44	0.537
KNN	63.7	0.75	0.601	0.885	0.716	0.299	0.250	2.40	1.47
DT	52.5	0.459	0.581	0.565	0.573	0.034	0.540	1.07	0.972
GBM	71.8	0.687	0.746	0.726	0.736	0.434	0.528	1.41	0.973
SVM	76.6	0.809	0.729	0.813	0.555	0.538	0.190	3.83	1.11
RF	73	0.6	0.8	0.8	0.8	0.4	0.400	2.00	1.00
PLSDA	54.9	0.431	0.602	0.702	0.648	0.212	0.568	1.05	1.16

**Table 1B:** Performance matrices of top genes predicted under biotic stress in maize.

Naive Bayes		GBM		DT		RF		SVM		K-NN		PLSDA	
Genes	AUC	Genes	rel.inf	Genes	rel.inf	Genes	AUC	Genes	RMSE	Genes	AUC	Genes	VIP-score
Zm00001eb034620	100	Zm00001eb024180	2.749	Zm00001eb110400	15.767	Zm00001eb088840	100	Zm00001eb019130	13.103	Zm00001eb034620	100	Zm00001eb176300	3.597
Zm00001eb019590	95.14	Zm00001eb034330	1.932	Zm00001eb087410	13.015	Zm00001eb034620	97.71	Zm00001eb034390	10.995	Zm00001eb145370	99.23	Zm00001eb021020	3.484
Zm00001eb136760	94.34	Zm00001eb164530	1.601	Zm00001eb097390	12.867	Zm00001eb158600	97.7	Zm00001eb090610	8.963	Zm00001eb034390	99.09	Zm00001eb047030	3.404
Zm00001eb071870	90.04	Zm00001eb071760	1.547	Zm00001eb034390	12.142	Zm00001eb090610	95.54	Zm00001eb181690	3.937	Zm00001eb070640	94.85	Zm00001eb038720	3.316
Zm00001eb145370	88.4	Zm00001eb068730	1.537	Zm00001eb088150	9.324	Zm00001eb034390	71.42	Zm00001eb088150	3.567	Zm00001eb037820	94.61	Zm00001eb168410	3.269
Zm00001eb150630	87.8	Zm00001eb029780	1.48	Zm00001eb160330	8.25	Zm00001eb042770	57.97	Zm00001eb115060	3.486	Zm00001eb166710	91.05	Zm00001eb137800	3.267
Zm00001eb067180	87.11	Zm00001eb084520	1.434	Zm00001eb019620	8.097	Zm00001eb151510	55.2	Zm00001eb110450	3.467	Zm00001eb063720	90.73	Zm00001eb100490	3.203
Zm00001eb103210	86.27	Zm00001eb046720	1.405	Zm00001eb171130	7.986	Zm00001eb010720	54.44	Zm00001eb184360	2.821	Zm00001eb090610	90.54	Zm00001eb096510	3.193
Zm00001eb050760	85.22	Zm00001eb027600	1.38	Zm00001eb034620	7.939	Zm00001eb078220	53.74	Zm00001eb100260	2.778	Zm00001eb150630	86.82	Zm00001eb022830	3.182
Zm00001eb157100	85.19	Zm00001eb103260	1.373	Zm00001eb053690	7.351	Zm00001eb002660	51	Zm00001eb109670	2.526	Zm00001eb028560	86.76	Zm00001eb143050	3.149
Zm00001eb143200	84.91	Zm00001eb075780	1.365	Zm00001eb034330	7.005	Zm00001eb144000	48.32	Zm00001eb146780	2.301	Zm00001eb067440	86.22	Zm00001eb056920	3.135
Zm00001eb097950	84.9	Zm00001eb156230	1.321	Zm00001eb034490	7.005	Zm00001eb088150	45.85	Zm00001eb167510	2.281	Zm00001eb043810	85.91	Zm00001eb111400	3.134
Zm00001eb156230	84.7	Zm00001eb061490	1.307	Zm00001eb043550	6.274	Zm00001eb143200	44.58	Zm00001eb167230	2.031	Zm00001eb020250	85.57	Zm00001eb065380	3.13
Zm00001eb088150	84.59	Zm00001eb155370	1.263	Zm00001eb015830	5.476	Zm00001eb063710	42.95	Zm00001eb097390	2.02	Zm00001eb071760	84.86	Zm00001eb016660	3.124
Zm00001eb002660	83.45	Zm00001eb133930	1.208	Zm00001eb016210	5.219	Zm00001eb094950	42.68	Zm00001eb020250	1.927	Zm00001eb099610	84.67	Zm00001eb081510	3.097
Zm00001eb110740	83.4	Zm00001eb003880	1.19	Zm00001eb018440	5.085	Zm00001eb019130	41.4	Zm00001eb088840	1.879	Zm00001eb075030	84.32	Zm00001eb098910	3.09
Zm00001eb182980	83.32	Zm00001eb083490	1.1849	Zm00001eb116160	5.085	Zm00001eb011650	39.35	Zm00001eb087410	1.842	Zm00001eb150350	84.28	Zm00001eb131030	3.089
Zm00001eb070860	83.29	Zm00001eb154470	1.182	Zm00001eb018350	4.87	Zm00001eb174340	39.35	Zm00001eb118770	1.74	Zm00001eb165610	83.8	Zm00001eb039370	3.087
Zm00001eb109860	83.07	Zm00001eb157410	1.175	Zm00001eb063710	4.662	Zm00001eb097390	37.74	Zm00001eb042770	1.587	Zm00001eb075420	83.65	Zm00001eb104430	3.087
Zm00001eb078870	82.68	Zm00001eb045900	1.166	Zm00001eb075030	4.662	Zm00001eb071400	37.58	Zm00001eb170900	1.555	Zm00001eb094950	83.55	Zm00001eb043420	3.085

MCC = Mathews correlation coefficient, ROC = receiver operating characteristic, PRC = precision recall curve.

The top 20 genes predicted by each model resulted in a total of 111 unique top significantly differentially regulated genes by all seven models (**Table 1B**). Of these, 16 genes were predicted by at least two models. Four genes, *Zm00001eb034390*, *Zm00001eb088150*, *Zm00001eb042770*, and *Zm00001eb097390* predicted as top genes by two or more models including the two high-performing models, SVM and RF, were considered the most significantly differentially expressed genes under biotic stress.

#### Prediction of top genes under abiotic stress

The confusion matrix (**Supplementary Table 2**) for the abiotic stress responsive genes revealed that the highest average accuracy of 88.6% was obtained by GBM, closely followed by SVM (87.5%) and RF (87.0%) algorithms (**Table 2A**). RF had the highest MCC value (0.72) followed by SVM at 0.71 and GBM (0.6). SVM had the highest specificity (0.94) followed by RF at 0.89 and precision (0.86). Altogether, 68 unique genes were identified as most informative by all seven models. Twenty-one genes were consistently predicted as top abiotic stress-related genes by at least two models of which 12 were common in four or more models, which included GBM, SVM, RF and KNN models that had higher accuracy and/or model performance matrices compared to other models (**Table 2B**). Interestingly, only one gene *Zm00001eb038720*, predicted by PLSDA, was consistent between the biotic and abiotic stress conditions.

**Table 2A:** Confusion matrices of machine learning algorithms used with gene expression values under abiotic stress in maize.

Models	Accuracy (%)	Specificity	Sensitivity	Precision	F1-score	MCC	FP Rate	ROC Area	PRC Area
NB	80.30	0.893	0.675	0.813	0.748	0.589	0.107	6.30	1.20
KNN	80.00	0.767	0.932	0.495	0.647	0.580	0.397	2.34	0.531
DT	77.33	0.801	0.721	0.657	0.688	0.511	0.198	3.64	0.911
GBM	88.60	0.895	0.868	0.803	0.834	0.600	0.104	8.34	0.925
SVM	87.50	0.939	0.750	0.864	0.803	0.716	0.058	12.93	1.15
RF	87.00	0.897	0.840	0.790	0.984	0.725	0.111	7.56	0.940
PLSDA	78.10	0.853	0.644	0.697	0.695	0.507	0.146	4.41	1.08

**Table 2B:** Performance matrices of top genes predicted under abiotic stress in maize.

Naive Bayes		GBM		DT		RF		SVM		K-NN		PLSDA	
Genes	AUC	Genes	rel.inf	Genes	rel.inf	Genes	AUC	Genes	RMSE	Genes	AUC	Genes	VIP- score
Zm00001eb012040	100	Zm00001eb146690	5.456	Zm00001eb012040	134.264	Zm00001eb112930	100	Zm00001eb012040	164.770	Zm00001eb160470	100	Zm00001eb133500	4.406
Zm00001eb146690	98.30	Zm00001eb058820	5.212	Zm00001eb112930	96.502	Zm00001eb021010	88.00	Zm00001eb160470	72.756	Zm00001eb176940	99.94	Zm00001eb027100	4.332
Zm00001eb176680	97.50	Zm00001eb012040	4.980	Zm00001eb160470	94.104	Zm00001eb012040	83.00	Zm00001eb176680	52.022	Zm00001eb012040	99.62	Zm00001eb130760	4.200
Zm00001eb021010	97.50	Zm00001eb044020	4.689	Zm00001eb098220	91.107	Zm00001eb176680	80.30	Zm00001eb201180	33.061	Zm00001eb126900	99.05	Zm00001eb117220	4.158
Zm00001eb160470	96.80	Zm00001eb176680	4.590	Zm00001eb050500	88.710	Zm00001eb146690	78.30	Zm00001eb146690	20.824	Zm00001eb050500	98.94	Zm00001eb099850	3.991
Zm00001eb044020	96.40	Zm00001eb176940	2.674	Zm00001eb179190	86.312	Zm00001eb058820	76.40	Zm00001eb248930	20.611	Zm00001eb044020	98.60	Zm00001eb104530	3.899
Zm00001eb112930	95.50	Zm00001eb021010	2.138	Zm00001eb151430	18.992	Zm00001eb160470	74.10	Zm00001eb192710	19.716	Zm00001eb176680	97.59	Zm00001eb068620	3.884
Zm00001eb050500	95.50	Zm00001eb075250	1.858	Zm00001eb072870	16.477	Zm00001eb044020	66.90	Zm00001eb238010	16.154	Zm00001eb179190	97.43	Zm00001eb090990	3.824
Zm00001eb176940	95.20	Zm00001eb030400	1.591	Zm00001eb065100	13.599	Zm00001eb098220	65.60	Zm00001eb203690	15.104	Zm00001eb146690	96.90	Zm00001eb115290	3.776
Zm00001eb151430	95.00	Zm00001eb151430	1.478	Zm00001eb156020	10.218	Zm00001eb176940	55.00	Zm00001eb050500	14.803	Zm00001eb151430	95.77	Zm00001eb149960	3.769
Zm00001eb058820	94.50	Zm00001eb156510	1.329	Zm00001eb171000	8.514	Zm00001eb018700	53.50	Zm00001eb176940	14.077	Zm00001eb041030	95.20	Zm00001eb037980	3.763
Zm00001eb179190	92.90	Zm00001eb119820	1.320	Zm00001eb143640	7.682	Zm00001eb076550	51.80	Zm00001eb021010	10.680	Zm00001eb076550	95.17	Zm00001eb046590	3.754
Zm00001eb126900	92.10	Zm00001eb050500	1.267	Zm00001eb076250	7.622	Zm00001eb151430	50.10	Zm00001eb250120	8.224	Zm00001eb058820	95.05	Zm00001eb000340	3.747
Zm00001eb041030	91.10	Zm00001eb074930	0.945	Zm00001eb019280	7.414	Zm00001eb179190	49.80	Zm00001eb017550	7.669	Zm00001eb112930	94.89	Zm00001eb114240	3.745
Zm00001eb098220	90.50	Zm00001eb057510	0.939	Zm00001eb136970	6.664	Zm00001eb078640	47.60	Zm00001eb112930	7.260	Zm00001eb021010	94.61	Zm00001eb038720	3.726
Zm00001eb018700	90.10	Zm00001eb171000	0.903	Zm00001eb154950	6.590	Zm00001eb050500	45.50	Zm00001eb189080	5.677	Zm00001eb005840	94.24	Zm00001eb066950	3.687
Zm00001eb117180	89.80	Zm00001eb111020	0.889	Zm00001eb013080	6.219	Zm00001eb148130	38.10	Zm00001eb044020	5.583	Zm00001eb124290	92.63	Zm00001eb148300	3.677
Zm00001eb005840	89.80	Zm00001eb015730	0.873	Zm00001eb074360	6.219	Zm00001eb040280	37.10	Zm00001eb151430	4.550	Zm00001eb018700	92.50	Zm00001eb026650	3.673
Zm00001eb076550	89.80	Zm00001eb080960	0.858	Zm00001eb127040	6.219	Zm00001eb117180	36.90	Zm00001eb043060	4.516	Zm00001eb013780	91.83	Zm00001eb124020	3.671
Zm00001eb124290	89.70	Zm00001eb154060	0.816	Zm00001eb128310	6.004	Zm00001eb179280	35.00	Zm00001eb243850	4.176	Zm00001eb075250	91.53	Zm00001eb128720	3.660

MCC, Mathews correlation coefficient; ROC, receiver operating characteristic; PRC, precision recall curve.

#### Prediction of top genes under combined stress conditions

When the genes responsive to biotic and/or abiotic stress conditions based on the datasets in the literature were combinedly used for prediction by seven models, RF outperformed others with the highest accuracy (81.7%) and other performance matrices except for sensitivity (0.75), which was behind SVM (0.80) and GBM (0.77) (**Table 3A**; **Supplementary Table 2**). SVM predicted the top 20 genes with the highest sensitivity, an accuracy of 81.0%, F1-score (0.77) and MCC (0.61) second to only RF. GBM also performed good with 79.0% accuracy, nearly equal specificity and same sensitivity as SVM. NB was the worst predictor algorithm with the lowest values recorded for accuracy as well as other parameters. A total of 83 unique top significant genes were reported for combined stress by the seven models of which 11 genes were commonly predicted by four or more models. Among biotic and combined stress conditions, 23 genes were found common. On the other hand, only two genes were found within biotic and combined stress conditions (**Table 3B**).

**Table 3A:** Confusion matrices of machine learning algorithms used with gene expression values under combined stress conditions in maize.

Models	Accuracy (%)	Specificity	Sensitivity	Precision	F1-score	MCC	FP Rate	ROC Area	PRC Area
NB	65.10	0.680	0.591	0.466	0.523	0.232	0.319	1.85	0.788
KNN	72.00	0.725	0.711	0.537	0.612	0.401	0.275	2.58	0.755
DT	73.40	0.779	0.672	0.689	0.658	0.453	0.220	3.05	1.02
GBM	79.00	0.814	0.772	0.753	0.763	0.584	0.185	4.17	0.975
SVM	81.00	0.815	0.803	0.746	0.773	0.612	0.184	4.36	0.929
RF	81.70	0.865	0.755	0.813	0.783	0.627	0.134	5.63	1.07
PLSDA	78.00	0.840	0.690	0.760	0.720	0.549	0.158	4.36	1.10

**Table 3B:** Performance matrices of top genes predicted under combined stress conditions in maize.

Naive Bayes		GBM		DT		RF		SVM		K-NN		PLSDA	
Genes	AUC	Genes	rel.inf	Genes	rel.inf	Genes	AUC	Genes	RMSE	Genes	AUC	Genes	VIP-score
Zm00001eb050500	100	Zm00001eb112930	3.789	Zm00001eb176940	142.216	Zm00001eb076550	100	Zm00001eb050500	2.783	Zm00001eb050500	100	Zm00001eb130760	1.935
Zm00001eb146690	99.32	Zm00001eb179190	3.317	Zm00001eb018700	108.111	Zm00001eb179190	99.66	Zm00001eb176680	2.383	Zm00001eb176940	95.78	Zm00001eb163120	1.931
Zm00001eb176940	98.20	Zm00001eb050500	3.109	Zm00001eb041030	105.736	Zm00001eb050500	96.40	Zm00001eb176940	2.372	Zm00001eb146690	94.84	Zm00001eb162400	1.926
Zm00001eb112930	96.84	Zm00001eb126900	3.048	Zm00001eb126900	105.736	Zm00001eb160470	95.19	Zm00001eb126900	2.240	Zm00001eb160470	93.99	Zm00001eb104530	1.925
Zm00001eb058820	94.47	Zm00001eb176680	2.789	Zm00001eb050500	103.741	Zm00001eb176940	92.86	Zm00001eb041030	2.228	Zm00001eb112930	92.88	Zm00001eb113530	1.924
Zm00001eb176680	93.17	Zm00001eb176940	2.208	Zm00001eb075230	99.466	Zm00001eb126900	85.99	Zm00001eb018700	2.224	Zm00001eb176680	92.58	Zm00001eb177670	1.922
Zm00001eb160470	92.55	Zm00001eb146690	1.650	Zm00001eb012040	25.994	Zm00001eb112930	80.01	Zm00001eb001220	1.902	Zm00001eb058820	89.88	Zm00001eb165700	1.921
Zm00001eb179190	90.05	Zm00001eb058820	1.326	Zm00001eb071400	21.751	Zm00001eb176680	73.21	Zm00001eb003440	1.900	Zm00001eb179190	88.72	Zm00001eb149960	1.921
Zm00001eb033200	87.67	Zm00001eb154060	1.006	Zm00001eb005840	21.107	Zm00001eb018700	73.05	Zm00001eb050470	1.870	Zm00001eb154060	88.22	Zm00001eb050770	1.920
Zm00001eb154060	87.42	Zm00001eb160470	0.982	Zm00001eb116880	20.899	Zm00001eb146690	65.41	Zm00001eb160470	1.667	Zm00001eb041030	86.33	Zm00001eb028190	1.918
Zm00001eb041030	87.23	Zm00001eb021720	0.841	Zm00001eb021010	20.275	Zm00001eb037690	63.90	Zm00001eb058820	1.649	Zm00001eb156130	84.24	Zm00001eb028490	1.918
Zm00001eb018180	85.09	Zm00001eb054980	0.833	Zm00001eb031210	20.275	Zm00001eb058820	62.44	Zm00001eb146690	1.569	Zm00001eb146500	81.84	Zm00001eb089070	1.918
Zm00001eb156130	84.54	Zm00001eb042770	0.828	Zm00001eb155430	20.067	Zm00001eb012040	60.49	Zm00001eb093590	1.451	Zm00001eb038010	81.69	Zm00001eb138650	1.917
Zm00001eb138530	83.53	Zm00001eb140230	0.772	Zm00001eb166600	17.528	Zm00001eb154060	59.43	Zm00001eb004630	1.443	Zm00001eb033200	81.28	Zm00001eb154420	1.917
Zm00001eb146500	83.38	Zm00001eb079740	0.714	Zm00001eb019280	16.114	Zm00001eb075230	58.23	Zm00001eb040610	1.434	Zm00001eb138530	81.13	Zm00001eb042970	1.917
Zm00001eb003440	83.01	Zm00001eb057750	0.686	Zm00001eb171940	14.569	Zm00001eb041030	46.05	Zm00001eb129480	1.410	Zm00001eb016200	80.71	Zm00001eb124300	1.917
Zm00001eb004630	82.89	Zm00001eb020980	0.671	Zm00001eb171930	14.341	Zm00001eb148130	45.43	Zm00001eb111990	1.398	Zm00001eb040610	80.69	Zm00001eb172930	1.916
Zm00001eb010960	82.43	Zm00001eb146390	0.670	Zm00001eb166670	14.114	Zm00001eb179280	42.10	Zm00001eb098220	1.363	Zm00001eb179280	80.67	Zm00001eb033410	1.916
Zm00001eb014630	82.43	Zm00001eb016200	0.649	Zm00001eb166610	13.886	Zm00001eb020260	41.89	Zm00001eb101050	1.354	Zm00001eb078640	80.29	Zm00001eb042710	1.916
Zm00001eb163520	82.41	Zm00001eb102720	0.642	Zm00001eb170070	13.886	Zm00001eb050850	38.64	Zm00001eb112930	1.349	Zm00001eb014630	80.08	Zm00001eb131600	1.916

MCC, Mathews correlation coefficient; ROC, receiver operating characteristic; PRC, precision recall curve.

Taken together, SVM, RF, and GBM were identified as the best models in predicting top significant (a)biotic stress responsive genes with high accuracy in our study. However, Nazari et al. (2023) found KNN (82.0%) and Ensemble (85.7%) to be more accurate for predicting biotic stress tolerance genes while modeling gene expression data from microarray studies in maize. Interestingly, none of the significant genes identified by these authors matched the top genes selected by the seven models used in our study.

### Significant modules and potential hub genes

**Figure 1:**
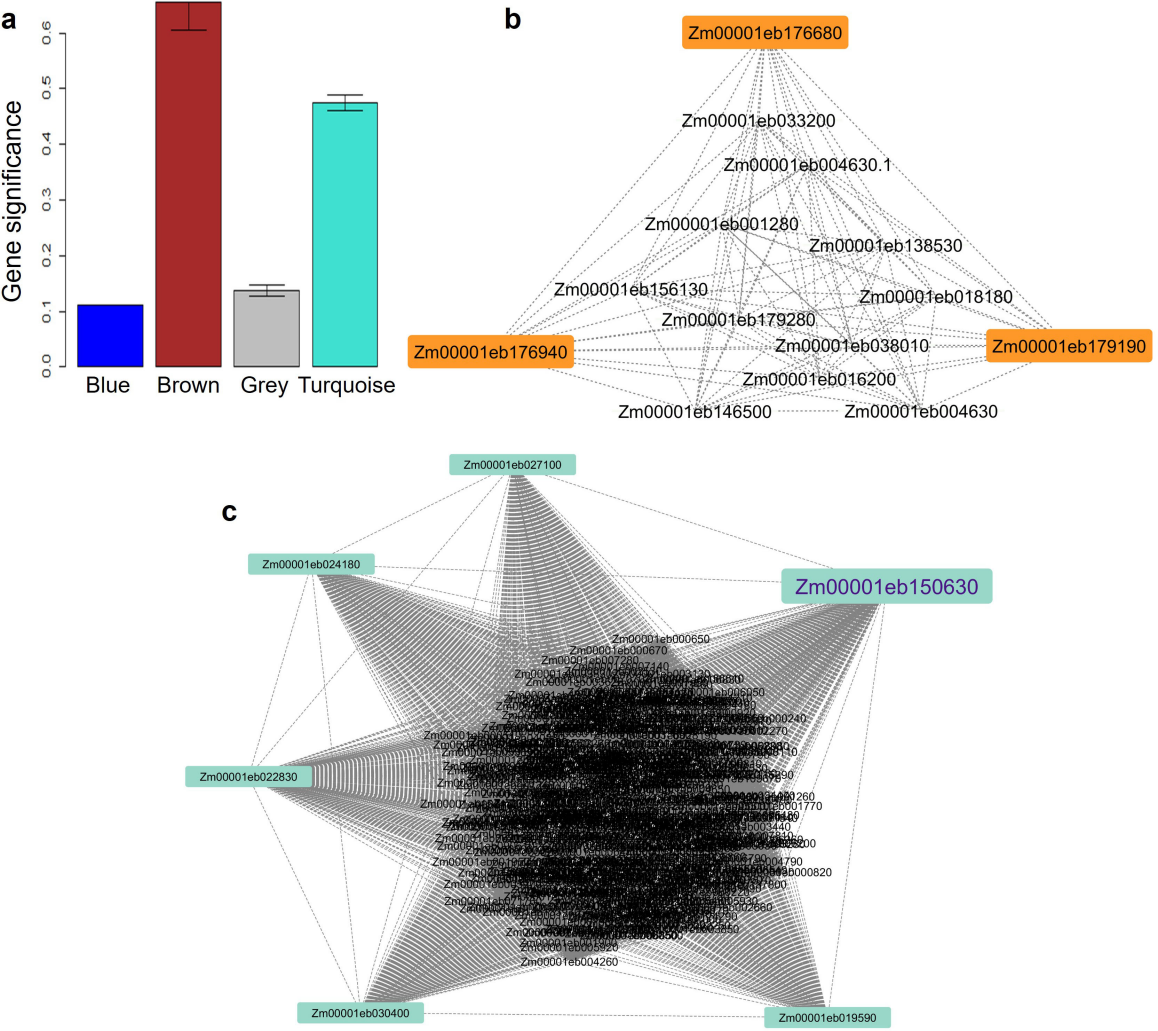
The gene significance values (*P-*value=8.9e-124) across co-expression network modules **(a)**. Brown **(b)** and Turquoise **(c)** significant modules showing three and six potential hub genes, respectively.

The weighted gene co-expression network analysis based on a height cut-off at 0.25 to merge the modules, detected four modules of which Turquoise and Brown modules with 438 and 14 genes, respectively were significant with r >0.8 and GS (P-value¾0.01) (**Supplementary Table 3**; **Figure 1a**). Three significant hub genes, *Zm00001eb176680, Zm00001eb176940*, and *Zm00001eb179190* with KME>0.8, were identified in the brown module (**Figure 1b**) for abiotic stress and one gene *Zm00001eb150630* (turquoise model) for biotic stress (**Figure 1c**) that were predicted by two or more models.

### Functional involvement of the top highly significant genes

GO analysis of top genes representing conserved up and down-regulation under stress conditions identified 47 and 3 significant GO terms (*p*-value<0.001; FDR <0.05) for abiotic and biotic stress, respectively whereas 17 significant GO terms were identified for combined stress (**Supplementary Table 4**). The most significantly enriched molecular function with the highest number of genes was associated with nucleotide binding for biotic stress whereas binding followed by response to stimulus were the most enriched processes under abiotic stress. On the other hand, cell communication, cellular response to alcohol, lipid, external stimuli, and abscisic acid, and hormone signaling were the two most enriched biological processes under combined stress conditions. The biological pathways involving the top-most significant genes involved are presented in **Supplementary Table 5**. In biotic stress, genes involved in calcium ion binding were the most enriched whereas genes in abscisic acid activated signaling pathway, cation binding and myosin phosphate activity were the most significant in abiotic stress. Genes associated with seed nutrient storage activity, cation binding, and plant hormone signal transduction were significant under combined, biotic and abiotic stresses.

Of the four top-most significant genes associated with biotic stress response and predicted by the two high-performing models SVM and RF, *Zm00001eb034390* (GO:0005509) coding for EF-hand 1 calcium binding protein (1.23-fold), *Zm00001eb088150* (GO:0098754) for UDP-glucuronosyl/UDP-glucosyltransferase (0.74-fold), *Zm00001eb042770* (GO:0005634) for ribonuclease 3-like protein 3 (0.23) were significantly upregulated, whereas gene *Zm00001eb097390* (GO:0006457) encoding GrpE nucleotide exchange factor was downregulated (-0.14) under fungal infection (**Supplementary Table 6**) (Shu et al., 2017; Kebede et al., 2018; Shi et al., 2018; Han et al., 2020; Lambarey et al., 2020; Musungu et al., 2020; He et al., 2021; Liu et al., 2021; Schurack et al., 2021). *Zm00001eb088150* has been shown to be involved in the detoxification of several exogenous and endogenous compounds, and it plays multiple roles in plant responses to biotic as well as abiotic stresses (Gharabli et al., 2023) providing protection against mycotoxins (Wetterhorn, 2018), pathogens, drought, heat, cold, and salinity (Van Aken, 2008; Meale et al., 2015). UDP-glucuronosyl/UDP-glucosyltransferase was found to be one of the 26 genes commonly regulated between maize, peanut, and cotton in response to *Aspergillus flavus*, and it was upregulated in both pericarp and seed tissues of cotton (Mehanathan et al., 2018).

Among the 12 most-significant genes predicted by SVM, RF, and GBM for abiotic stress, eight were upregulated under drought (Kakumanu et al., 2012; Song et al., 2017; Li et al., 2017; Thirunavukkarasu et al., 2017; SkZ et al., 2018; Yang et al., 2019; Danilevskaya et al., 2019; Zenda et al., 2019; Jia et al., 2020; Li et al., 2021; Kim et al., 2021; Maheswari et al., 2021; Bai et al., 2022), cold (Sobkowiak et al., 2014; Mao et al., 2017; Goering, 2017; Li et al., 2017; Avila et al., 2018; Waititu et al., 2021; Li et al., 2021), heat (Li et al., 2014; Shi et al., 2017; Li et al., 2017 and Zhao et al., 2019), and salt (Zhang et al., 2015; Du et al., 2017; Li et al., 2017; Chen et al., 2020; Zhang et al., 2021; 2022) stress of which four were downregulated under waterlogging condition (**Supplementary Table 6**) (Arora et al., 2017; Yao, 2021). Also, eight of the genes were commonly predicted between abiotic and combined stresses. Of the four genes specifically uniquely predicted for abiotic stress, *Zm00001eb012040* (GO:0006470) is expressed as a PPM-type phosphatase protein and was upregulated in all but waterlogging conditions whereas *Zm00001eb160470*, which also codes for a protein phosphatase, was upregulated under drought, heat and salt but downregulated under cold and waterlogging conditions (**Supplementary Table 6**). These genes are known to dephosphorylate serine/threonine in stress responsive genes in maize, thus impacting its response to multiple stresses (drought, salt) via hormone signal transductions (He et al., 2019). On the other hand, *Zm00001eb021010* (cysteine-rich and transmembrane domain-containing protein WIH1) was downregulated in all abiotic stresses except slight upregulation under waterlogging conditions. Several members of the cysteine-rich peptide family have been shown to respond extensively to various abiotic stresses in different plants including maize (Xu et al., 2018). *Zm00001eb050500* (GO:0009653), a top gene under abiotic as well as combined stress, encodes expansin, which plays an important role in stress relaxation of (arabino) xylan-cellulose networks within the cell wall (Yennawar et al., 2006). bZip-transcription factors (e.g., *Zm00001eb058820*) have been extensively studied for their critical roles in regulating plants response to abiotic stresses through morphological adaptations (Guo et al., 2024). In maize, a member of bZIP family positive regulated stress resistance through ABA-dependent signaling (He et al., 2024). *Zm00001eb044020* (a P-loop containing nucleoside triphosphate hydrolase), which was moderately upregulated under cold and waterlogging conditions, but highly upregulated in response to drought, heat and salt, was also identified to be induced by proline under low water potential (Verslues et al., 2014) and was located within the genomic region associated with heat stress (Bashir et al., 2025). Only one gene *Zm00001eb038720*, expressed as Ribonuclease E/G, was found commonly predicted between biotic and abiotic stress, although it was not identified as one of the top-most significant genes by the two or more high-performing accurate models. Ribonuclease E/G are chloroplastic endoribonuclease that play crucial roles in plant’s response to biotic and abiotic stresses because of their involvement in cleavage-mediated RNA homeostasis (Schein et al., 2008). The RNase E/G enzymes influence RNA modifications in plant adaptation to stresses by controlling mRNA stability and subsequent translation of genes (Cai et al., 2025).

Three genes that were commonly predicted as top-most candidates between abiotic stress and combined stresses as well as identified as the hub genes in the brown module, coded for a *bZIP* transcription factor 68 (*Zm00001eb176680*), glycine-rich cell wall structural protein 2 (*Zm00001eb176940*), and aldehyde dehydrogenase 11 (*ALDH11; Zm00001eb179190*). *bZIP* transcription factor 68 showed a negative response to cold stress in transgenic maize plants (Zeng et al., 2021). bZIP68 interacts with mitogen-activated protein kinase 8, which is also a negative regulator of the cold-stress response. A 358-bp indel in the *bZIP68* promoter region increased its expression resulting in decreased cold tolerance in maize (Li et al., 2022). *ZmbZIP4* was also shown to be differentially stimulated by high salinity, drought, heat, cold, and abscisic acid treatment in maize seedlings (Ma et al., 2018). Glycine-rich cell wall structural proteins (GRCWSPs) are known to be involved in plant’s response to abiotic stress, especially under osmotic stress because of their roles in maintaining cell wall integrity during dehydration by connecting the lignin rings to strengthen the cell wall (Ryser et al., 2004). The GRCWSPs interact with cell-wall associated kinases to initiate recognition of environmental stimuli and subsequent signal transduction (Park et al., 2001). *ALDH11* showed upregulation under drought, cold, heat and salt and negative regulation under waterlogging conditions. Several *ALDH* genes have been reported to contribute to improving salt and drought tolerance in plants (Wang et al., 2024).

### Promoter motifs in the top significant genes

Promoter regions corresponding to the top-most significantly predicted genes for different stresses showed 19 different CREs. As expected of the promoters, CAAT-box, TATA-box, and Unnamed_4 motifs existed in all promoters in high numbers (**Supplementary Table 7**). While there was no overall pattern in the distribution of the CREs among the promoters of the genes, the top biotic stress responsive genes, except *Zm00001eb042770*, had less overall total CREs. For most of the abiotic and combined stress responsive genes, the number of antioxidant responsive element (ARE) and ABRE (abscisic acid responsive element) were higher than the biotic stress responsive genes. The number of CREs were higher for two genes *Zm00001eb176940* (168) and *Zm00001eb179190* (169) predicted common between abiotic and combined stresses than the other genes. The ABRE and G-Box were found in more abundance in genes *Zm00001eb012040*, *Zm00001eb058820*, *Zm00001eb112930*, *Zm00001eb018700*, *Zm00001eb126900*, *Zm00001eb018700*, and *Zm00001eb126900* that were identified as top-most significant genes for abiotic and combined stresses.

## Conclusion

The integration of a large volume of gene expression data from several RNA-seq studies with machine learning methods increased the generalizability and statistical power and allowed us to analyze the stress responses of the genes to identify a set of top-most genes with significant associations with (a)biotic stress in maize. The GO and KEGG pathway enrichment analysis of top genes provided clues to the mechanisms underlying maize’s response to both biotic and abiotic stress conditions. Furthermore, the randomization procedure used in WGCNA method led to the identification of the hub genes validating their gene connectivity and possible interaction with other genes. Some of the genes identified in this study through ML were also found related to *Aspergillus flavus* resistance in maize in previous studies. Further functional validation of the roles of the 19 unique top-ranked genes including hub genes, predicted by the high-performing models, will lead to their utilization in developing multiple stress-resistant maize varieties.

## Data Availability

The original contributions presented in the study are included in the article/**Supplementary Material**. Further inquiries can be directed to the corresponding author/s.
